# Methodology for biomarker discovery with reproducibility in microbiome data using machine learning

**DOI:** 10.1186/s12859-024-05639-3

**Published:** 2024-01-15

**Authors:** David Rojas-Velazquez, Sarah Kidwai, Aletta D. Kraneveld, Alberto Tonda, Daniel Oberski, Johan Garssen, Alejandro Lopez-Rincon

**Affiliations:** 1 Division of Pharmacology, Utrecht Institute for Pharmaceutical Sciences, Faculty of Science, University of Utrecht, Utrecht, The Netherlands; 2 Department of Data Science, Julius Center for Health Sciences and Primary Care, University Medical Center Utrecht, Utrecht, The Netherlands; 3 Department of Neuroscience, Faculty of Science, Vrije Universiteit Amsterdam, Amsterdam, The Netherlands; 4 UMR 518 MIA - PS, INRAE, Institut des Systèmes Complexes de Paris, Île - de - France (ISC-PIF) - UAR 3611 CNRS, Université Paris-Saclay, Paris, France; 5 Global Centre of Excellence Immunology, Danone Nutricia Research, Utrecht, The Netherlands

**Keywords:** Machine learning, Reproducibility, Microbiome

## Abstract

**Background:**

In recent years, human microbiome studies have received increasing attention as this field is considered a potential source for clinical applications. With the advancements in omics technologies and AI, research focused on the discovery for potential biomarkers in the human microbiome using machine learning tools has produced positive outcomes. Despite the promising results, several issues can still be found in these studies such as datasets with small number of samples, inconsistent results, lack of uniform processing and methodologies, and other additional factors lead to lack of reproducibility in biomedical research. In this work, we propose a methodology that combines the DADA2 pipeline for 16s rRNA sequences processing and the Recursive Ensemble Feature Selection (REFS) in multiple datasets to increase reproducibility and obtain robust and reliable results in biomedical research.

**Results:**

Three experiments were performed analyzing microbiome data from patients/cases in Inflammatory Bowel Disease (IBD), Autism Spectrum Disorder (ASD), and Type 2 Diabetes (T2D). In each experiment, we found a biomarker signature in one dataset and applied to 2 other as further validation. The effectiveness of the proposed methodology was compared with other feature selection methods such as K-Best with F-score and random selection as a base line. The Area Under the Curve (AUC) was employed as a measure of diagnostic accuracy and used as a metric for comparing the results of the proposed methodology with other feature selection methods. Additionally, we use the Matthews Correlation Coefficient (MCC) as a metric to evaluate the performance of the methodology as well as for comparison with other feature selection methods.

**Conclusions:**

We developed a methodology for reproducible biomarker discovery for 16s rRNA microbiome sequence analysis, addressing the issues related with data dimensionality, inconsistent results and validation across independent datasets. The findings from the three experiments, across 9 different datasets, show that the proposed methodology achieved higher accuracy compared to other feature selection methods. This methodology is a first approach to increase reproducibility, to provide robust and reliable results.

**Supplementary information:**

The online version contains supplementary material available at 10.1186/s12859-024-05639-3.

## Background

In literature, human microbiome studies have received increasing attention. This domain is considered a potential source for the diagnosis and development of new medical treatments [1]. Several studies aim to identify variations in the gut microbiome and potential biomarkers to diagnose diseases and disorders such as inflammatory bowel disease (IBD) [2–5], type 2 diabetes (T2D) [6–9], autism spectrum disorder (ASD) [10–13], and some types of cancer [14–17], among others. Microbiome studies have also been used to develop medical treatments and to analyze the responses from patients [18–21]. Microbiome analysis consists in sequencing the gene encoding 16s ribosomal RNA (rRNA) and compare it with known bacteria sequence databases to identify bacterial members of a microbial population [22]. Several software tools and pipelines are available for this process, such as QIIME2 [23], VSEARCH [24], DADA2 [25], Trimmomatic [26], mothur [27], and FLASH [28]. These software tools allow performing the quality analysis of 16s rRNA raw data (filtering, trimming, chimera removal, merge sequences, taxonomy assignment) to generate Operational Taxonomy Units (OTUs) or Amplicon Sequence Variants (ASVs) and performing statistical analysis on the resulting bacterial taxonomy and abundance.

With the advancements in omics technologies and AI, research focused on the search for potential biomarkers in the human microbiome using machine learning tools has increased, where the use of taxonomy-based feature selection is one of the most common approach [29]. Nowadays, it is common to find research works that aims to find relevant taxonomy-based features and use them as potential biomarkers to apply them in medical conditions such as ASD [30, 31], cardiovascular disease [32], T2D [33, 34], IBD [35–38], Parkinson [39], and also to analyze the effect of medical treatments [40, 41]. Despite the promising results, several issues can still be found in these studies:

- Datasets: high dimensional data with a small number of samples are common, usually because of the costs (time and money) associated with data collection from human participants. This causes machine learning models prone to overfitting and biased performance [42].
- Inconsistent results: Most of the studies use Operational Taxonomy Units (OTUs) in their experiments, and due to the limitations and their inability to be used in independent studies [43, 44], may be the reason for obtaining inconsistent results [29, 45, 46].
- Reproducibility: Several factors such as the lack of uniform processing methodologies, incomplete or erroneous descriptions of the simulations, incomplete or erroneous dataset documentation, which software version was used, incomplete documentation, or not having the code available for use are responsible for a lack of reproducibility in microbial research [45, 47].

The main objective in this work, is to address the lack of reproducibility by providing a methodology, that considers more than one dataset, that combines a DADA2-based pipeline for 16s rRNA sequences processing and the Recursive Ensemble Feature Selection (REFS) algorithm, previously used in [48]. This methodology also provides an approach to deal with high dimensional data with a small number of samples, inconsistent results, and the lack of uniform processing and analysis methodologies. The effectiveness of the proposed methodology was tested by comparing its results with different feature selection methods. Three experiments were performed analyzing microbiome data related to: Inflammatory Bowel Disease (IBD), Autism Spectrum Disorder (ASD), and Type 2 Diabetes (T2D). The results of these experiments provide valuable insights about the performance of the proposed methodology and its potential application in microbiome research. Further research is needed to confirm these findings and to explore their potential clinical applications.

## Results

### Autism spectrum disorder (ASD)

#### Raw data processing

The trimming parameters used for DADA2-based srcipt filtering process and the number of Amplicon Sequence Variants (ASVs) generated for each dataset were:

- David et al – parameter: trimLeft = 10, 2040 ASVs generated.
- PRJNA589343 – parameter: truncLen = c(250), 2040 ASVs generated.
- PRJNA578223 – parameter: truncLen = c(290,220), 18,758 ASVs generated.

#### Feature selection phase

**Fig. 1:**
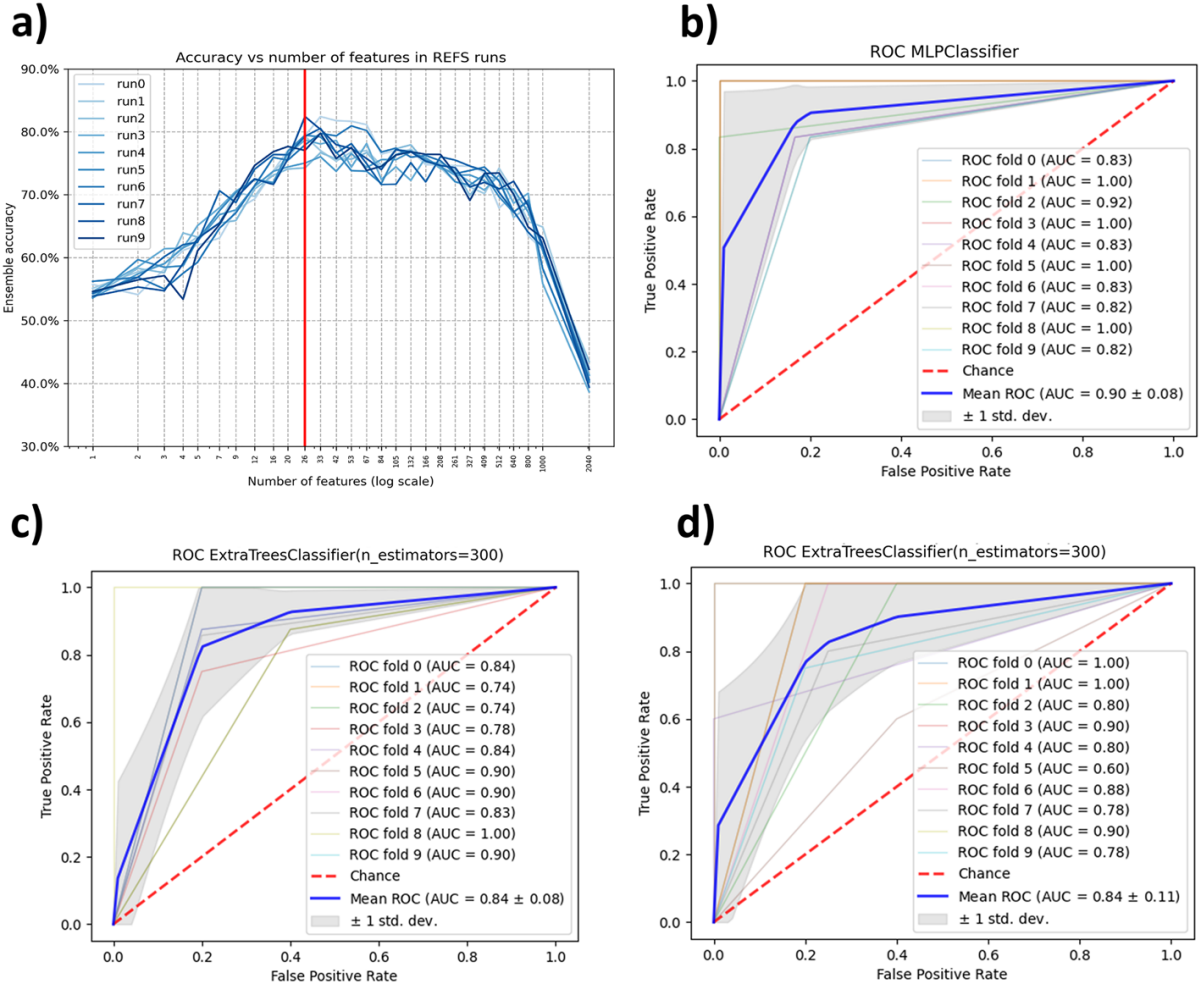
**a** The minimum number of features to obtain the higher accuracy, **b** Plot of the classifier with the best performance in the validation process for discovery dataset David et al, **c** Plot of the classifier with the best performance in the validation process for PRJNA589343, and **d** Plot of the classifier with the best performance in the validation process for PRJNA578223

We selected David et al [49] for discovery following the eligibility criteria. 26 out of the 2040 features resulted after applying the Recursive Ensemble Feature Selection (REFS) algorithm. This means, REFS achieved its highest accuracy (> 0.8) with 26 features, Fig. 1a. The result of the validation module for the selected 26 features was an average AUC of 0.816, which is considered “*very good*” diagnostic accuracy [50]. The Multilayer Perceptron (MLP) algorithm had the best performance, Fig. 1b.

In comparison, we applied the same validation module to the complete 2040 features, the resulting average AUC was 0.41. For feature selection using K-Best, with k = 26, the average AUC was 0.706. The detailed validation results are presented in Table 1. Using the Matthews correlation coefficient (MCC) as additional metric to evaluate the performance of the methodology, REFS achieved better average MCC (0.649) compared with the other feature selection methods, see Table 1.

**Table 1:** Individual and average AUCs and MCCs from the validation phase and the additional validation approaches applied to the ASD datasets. The standard deviation of each result was excluded to keep the table simple and avoid complexity

David et al.*	26 features (REFS)		2040 features		SelectKbest (k = 26)	
Classifier	AUC	MCC	AUC	MCC	AUC	MCC
AdaBoostClassifier	0.7200	0.4355	0.3900	− 0.1726	0.7500	0.5133
Extra Trees	0.7800	0.5934	0.3400	− 0.3664	0.7400	0.5195
KNeighbors	0.7900	0.6407	0.4200	0.0486	0.6200	0.2468
MLP	0.9000	0.8549	0.4100	− 0.0709	0.7500	0.3934
Lasso CV	0.8900	0.7207	0.5000	− 0.0447	0.6700	0.3177
**Average**	0.8160	0.6490	0.4100	− 0.1212	0.7060	0.3981

PRJNA589343	22 of 26 features (REFS)		SelectKbest (20 of 26 features)		10-time random selection	
Classifier	AUC	MCC	AUC	MCC	AUC	MCC
AdaBoostClassifier	0.7700	0.5968	0.6460	0.5139	0.7700	0.2474
Extra Trees	0.8400	0.7208	0.6510	0.6919	0.8000	0.3205
KNeighbors	0.6800	0.4808	0.6220	0.3967	0.6800	0.2636
MLP	0.7400	0.5119	0.6490	0.4181	0.7300	0.2863
Lasso CV	0.7100	0.0867	0.5710	0.1877	0.5400	0.1320
**Average**	0.7480	0.4794	0.6278	0.4416	0.7040	0.2500

PRJNA578223	20 of 26 features (REFS)		SelectKbest (17 of 26 features)		10-time random selection	
Classifier	AUC	MCC	AUC	MCC	AUC	MCC
AdaBoostClassifier	0.8300	0.7089	0.6530	0.2725	0.6700	0.3359
Extra Trees	0.8400	0.7105	0.6510	0.3877	0.6900	0.3578
KNeighbors	0.7000	0.2570	0.6370	0.2924	0.5900	0.3318
MLP	0.7200	0.4816	0.6120	0.4576	0.7300	0.2398
Lasso CV	0.6100	0.3779	0.6230	0.5025	0.7100	0.2738
**Average**	0.7400	0.5071	0.6352	0.3825	0.6780	0.3078

*Discovery dataset

#### Testing phase

We searched the 26 features selected by REFS in the testing datasets, the result was 22 out of 26 for PRJNA589343 and 20 out of 26 for PRJNA578223. We applied the validation module to the features found in both testing datasets. For PRJNA589343 we obtained an average AUC of 0.748 and for PRJNA578223 we obtained an average AUC of 0.74. Both average AUCs corresponds to a “*good*” diagnostic accuracy [50]. In both cases, the classifier with the best performance was Extra Trees, Fig. 1c,d.

For the comparative analysis, we searched for the 26 features selected by K-Best on each testing dataset, the result was 20 out of 26 for PRJNA589343 and 17 out of 26 for PRJNA578223. We applied the validation module to the features found in both testing datasets. The resulting average AUCs were 0.704 for PRJNA589343 and 0.678 for PRJNA578223. For the 10-time random selection the resulting average AUCs were 0.6278 for PRJNA589343 and 0.6352 for PRJNA578223. The detailed validation results are presented in Table 1. Using the MCC as additional metric for this phase, REFS achieved better performance in both testing datasets with average MCC values of 0.4794 for PRJNA589343 and 0.5071 for PRJNA578223, see Table 1.

### Inflammatory bowel disease (IBD)

#### Raw data processing

The trimming parameters used for DADA2-based srcipt filtering process and the number of Amplicon Sequence Variants (ASVs) generated for each dataset were:

- PRJEB21504 – parameter: trim = 20 and truncLen = c(160), 1793 ASVs generated.
- DRA006094 – parameter: trim = 20 and truncLen = c(200), 375 ASVs generated.
- PRJNA684584 – parameter: trim = 20, 1621 ASVs generated.

#### Feature selection phase

**Fig. 2:**
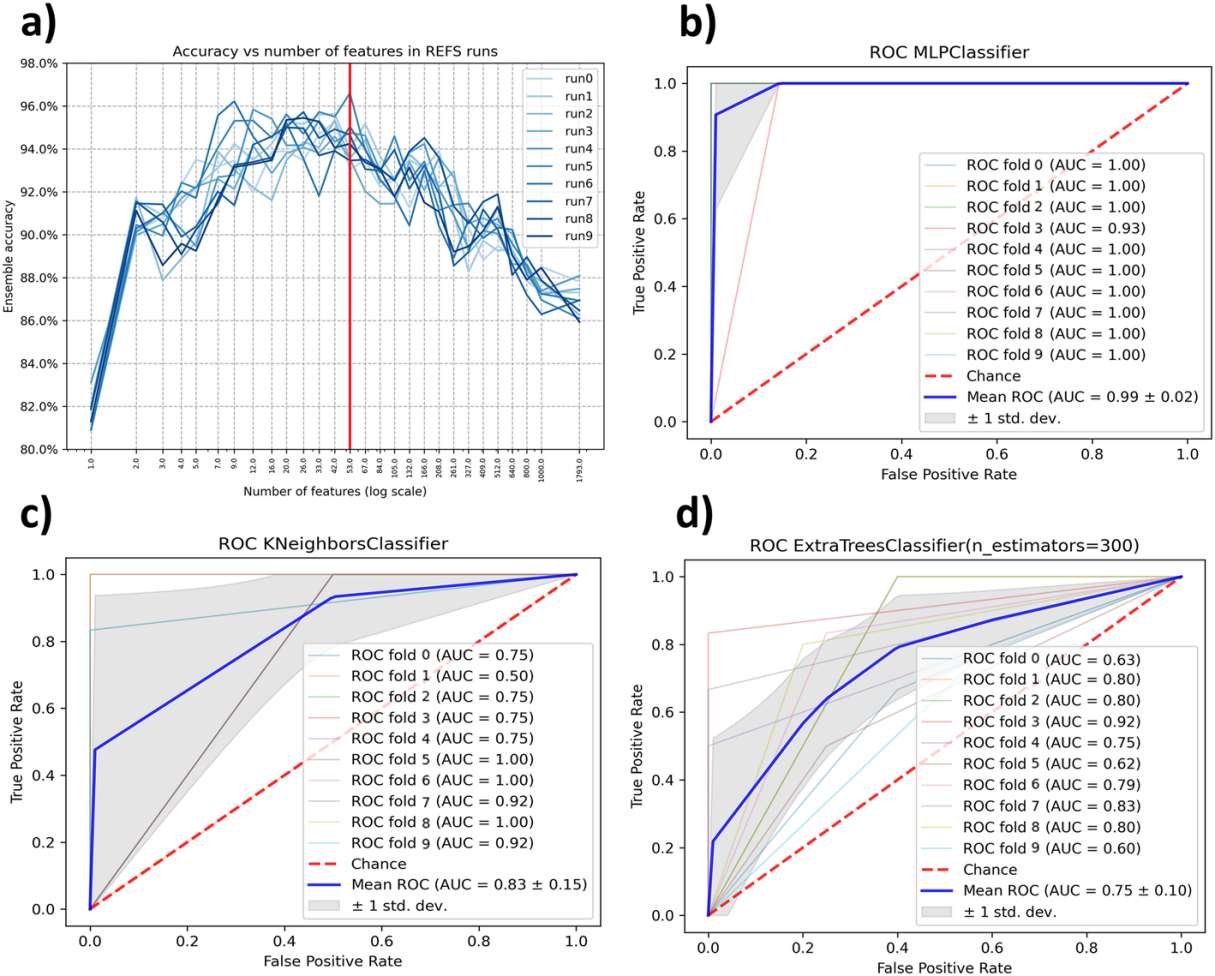
**a** The minimum number of features to obtain the higher accuracy, **b** Plot of the classifier with the best performance in the validation process for discovery dataset PRJEB2150, **c** Plot of the classifier with the best performance in the validation process for DRA00609, and **d** Plot of the classifier with the best performance in the validation process for PRJNA684584

We selected PRJEB21504 for discovery following the eligibility criteria. 53 out of the 1793 features resulted after applying the Recursive Ensemble Feature Selection (REFS) algorithm. This means, REFS achieved its highest accuracy (> 0.95) with 53 features, Fig. 2a. The result of the validation module for the selected 53 features was an average AUC of 0.936, considered “*excellent*” diagnostic accuracy [50]. The Multilayer Perceptron (MLP) algorithm had the best performance, Fig. 2b.

In contrast, we applied the same validation module to the complete 1793 features, the resulting average AUC was 0.718. For feature selection using K-Best, with k = 53, the average AUC was 0.902. The detailed validation results are presented in Table 2. Considering the Matthews correlation coefficient (MCC) as additional metric to evaluate the performance of the methodology, REFS achieved an average MCC value of 0.8715 which is higher than MCC values achieved by the other feature selection methods, see Table 2.

**Table 2:** Individual and average AUCs and MCCs from the validation phase and the additional validation approaches applied to the IBD datasets. The standard deviation of each result was excluded to keep the table simple and avoid complexity

PRJEB21504*	53 features (REFS)		1793 features		SelectKbest (k = 53)	
Classifier	AUC	MCC	AUC	MCC	AUC	MCC
AdaBoostClassifier	0.9100	0.8623	0.9100	0.8337	0.9100	0.8577
Extra Trees	0.9000	0.8841	0.8600	0.8049	0.9400	0.8577
KNeighbors	0.9300	0.8547	0.5400	0.0845	0.8900	0.8353
MLP	0.9900	0.9900	0.6100	0.2640	0.8900	0.8767
Lasso CV	0.9500	0.7564	0.6700	0.4064	0.8800	0.7165
**Average**	0.9360	0.8715	0.7180	0.4787	0.9020	0.8287

DRA006094	22 of 53 features (REFS)		SelectKbest (21 of 53 features)		10-time random selection	
Classifier	AUC	MCC	AUC	MCC	AUC	MCC
AdaBoostClassifier	0.7100	0.3087	0.5190	0.3288	0.7800	0.0761
Extra Trees	0.7800	0.4585	0.5210	0.3881	0.7200	0.0599
KNeighbors	0.8300	0.4245	0.5260	0.4070	0.6800	0.0093
MLP	0.8300	0.4418	0.5510	0.3881	0.7200	0.0916
Lasso CV	0.7400	0.3952	0.5230	0.4151	0.7600	0.0433
**Average**	0.7780	0.4057	0.5280	0.3854	0.7320	0.0560

PRJNA684584	48 of 53 features (REFS)		SelectKbest (52 of 53 features)		10-time random selection	
Classifier	AUC	MCC	AUC	MCC	AUC	MCC
AdaBoostClassifier	0.7200	0.4151	0.5410	0.3364	0.6400	0.1112
Extra Trees	0.7500	0.4300	0.5600	0.4026	0.7400	0.1612
KNeighbors	0.7000	0.3111	0.5610	0.1081	0.5900	0.1392
MLP	0.6800	0.3657	0.5700	0.2694	0.6800	0.1393
Lasso CV	0.7000	0.2616	0.5590	0.2908	0.6100	0.1420
**Average**	0.7100	0.3567	0.5582	0.2814	0.6520	0.1386

*Discovery dataset

#### Testing phase

We searched the 53 features selected by REFS in each testing dataset, the result was 22 out of 53 for DRA006094 and 48 out of 53 for PRJNA684584. After applying the validation module, we obtained an average AUC of 0.778 for DRA006094 and for PRJNA684584 we obtained an average AUC of 0.71. Both average AUCs correspond to a “*good*” diagnostic accuracy [50]. In this case, the classifier with the best performance was KNeighbors for DRA006094 and Extra Trees for PRJNA684584, Fig. 2c,d.

For the comparative analysis, we searched for the 53 features selected by K-Best on the testing datasets. The result was 21 out of 53 for DRA006094 and 52 out of 53 for PRJNA684584. We applied the validation module to the features found in both testing datasets. The resulting average AUCs were 0.732 for DRA006094 and 0.652 for PRJNA684584. For the 10-time random selection the resulting average AUCs were 0.528 for DRA006094 and 0.5582 for PRJNA684584. The detailed validation results are presented in Table 2. Using the MCC as additional metric for this phase, REFS achieved better performance in both testing datasets with average MCC values of 0.4057 for DRA006094 and 0.3567 for PRJNA684584, see Table 2.

### Type 2 diabetes (T2D)

#### Raw data processing

The trimming parameters used for DADA2-based srcipt filtering process and the number of Amplicon Sequence Variants (ASVs) generated for each dataset were:

- PRJNA3259311 – parameter: trimLeft = 15, 3316 ASVs generated.
- PRJNA5545355 – parameter: truncLen = c(400), 3201 ASVs generated.
- PRJEB53017 - no parameter used, 3672 ASVs generated.

#### Feature selection phase

**Fig. 3:**
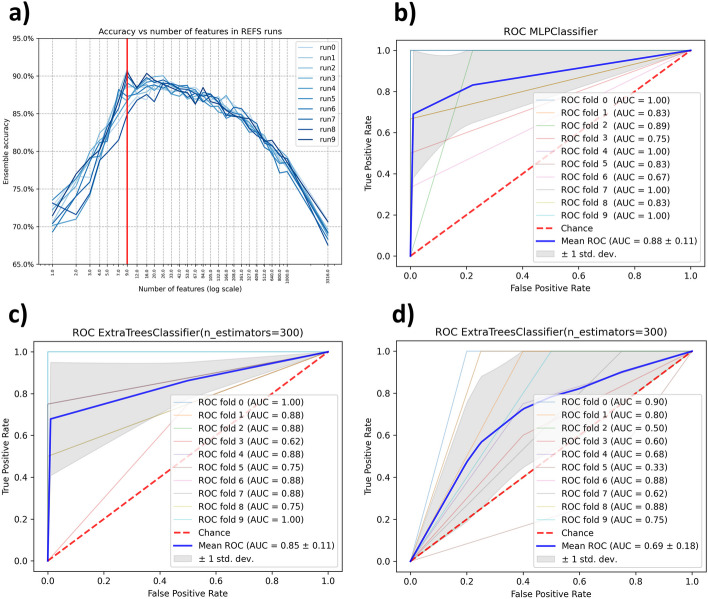
**a** The minimum number of features to obtain the higher accuracy, **b** Plot of the classifier with the best performance in the validation process for discovery dataset PRJNA325931, **c** Plot of the classifier with the best performance in the validation process for PRJNA554535, and **d** Plot of the classifier with the best performance in the validation process for PRJEB53017

We selected PRJNA3259311 for discovery according to the eligibility criteria. 9 out of the 3316 features resulted by using the Recursive Ensemble Feature Selection (REFS) algorithm. Thus, REFS achieved its highest accuracy (> 0.90) with 9 features, Fig. 3a. The result of the validation module for the selected 9 features was an average AUC of 0.79, which is considered “*good*” diagnostic accuracy [50]. In this case, the Multilayer Perceptron (MLP) the algorithm had the best performance, Fig. 3b.

In comparison, we applied the same validation module to the total 3316 features, the resulting average AUC was 0.494. For feature selection using K-Best, with k = 9, the average AUC was 0.75. The detailed validation results are presented in Table 3. Using the Matthews correlation coefficient (MCC) as additional metric to evaluate the performance of the methodology, REFS achieved better performance, compared with the other feature selection methods, with an average MCC of 0.79, see Table 3.

#### Testing phase

We searched the 9 features selected by REFS in each testing dataset, the result was 5 out of 9 for both testing datasets. We applied the validation module to the features found in both testing datasets. For PRJNA5545355 we obtained an average AUC of 0.714 and for PRJEB53017 we obtained an average AUC of 0.662. The average AUC for PRJNA5545355 corresponds to a “*good*” diagnostic accuracy and for PRJEB53017 the average AUC corresponds to a “*sufficient*” [50]. For both testing datasets, the classifier with the best performance was Extra Trees, Fig. 3c, d.

For the comparative analysis, we searched for the 9 features selected by K-Best on each testing dataset, the result was 4 out of 9 for both testing datasets. We applied the validation module to the features found in both testing datasets. The resulting average AUCs were 0.668 for PRJNA5545355 and 0.582 for PRJEB53017. For the 10-time random selection the resulting average AUCs were 0.5238 for PRJNA5545355 and 0.5154 for PRJEB53017. The detailed validation results are presented in Table 3. Using the MCC as additional metric for this phase, REFS achieved better performance in both testing datasets with average MCC values of 0.4210 for PRJNA5545355 and 0.3429 for PRJEB53017, see Table 3.

**Table 3:** Individual and average AUCs and MCCs from the validation phase and the additional validation approaches applied to the T2D datasets. The standard deviation of each result was excluded to keep the table simple and avoid complexity

PRJNA325931*	9 features (REFS)		3316 features		SelectKbest (k = 9)	
Classifier	AUC	MCC	AUC	MCC	AUC	MCC
AdaBoostClassifier	0.8000	0.6749	0.4500	0.0438	0.7600	0.4530
Extra trees	0.8400	0.7532	0.5000	0.0000	0.7600	0.5512
KNeighbors	0.6500	0.4033	0.5000	− 0.0428	0.6500	0.2319
MLP	0.8800	0.8064	0.5200	− 0.0083	0.8200	0.6792
Lasso CV	0.7800	0.5661	0.5000	0.1828	0.7600	0.5758
**Average**	0.7900	0.6407	0.4940	0.0351	0.7500	0.4982

PRJNA554535	5 of 9 features (REFS)		SelectKbest (4 of 9 features)		10-time random selection	
Classifier	AUC	MCC	AUC	MCC	AUC	MCC
AdaBoostClassifier	0.8200	0.6090	0.5260	0.5800	0.8000	0.0525
Extra Trees	0.8500	0.6504	0.5310	0.6093	0.8000	0.0684
KNeighbors	0.6700	0.3840	0.5230	0.4984	0.7300	0.0374
MLP	0.7100	0.4765	0.5230	0.3952	0.6000	0.0600
Lasso CV	0.5200	− 0.0146	0.5160	− 0.0158	0.5100	0.0296
**Average**	0.7140	0.4210	0.5238	0.4134	0.6880	0.0496

PRJEB53017	5 of 9 features (REFS)		SelectKbest (4 of 9 features)		10-time random selection	
Classifier	AUC	MCC	AUC	MCC	AUC	MCC
AdaBoostClassifier	0.6700	0.3036	0.5200	0.0425	0.5500	0.0517
Extra Trees	0.6900	0.3659	0.5230	0.2526	0.6000	0.0550
KNeighbors	0.6800	0.4124	0.4970	0.2977	0.6200	−0.0164
MLP	0.6600	0.3823	0.5270	0.0711	0.5400	0.0741
Lasso CV	0.6100	0.2505	0.5100	0.2035	0.6000	0.0189
**Average**	0.6620	0.3429	0.5154	0.1734	0.5820	0.0366

*Discovery dataset

### Discussion

In traditional analyses, groups of taxa called Operational Taxonomy Units (OTUs) are generated with sequences that are similar with a percentage of error, usually 3% [43, 44]. Considering this error, it is possible to miss variations (possible mutations) making a specific taxa that could be important in medical applications unable to be analyzed. Using Amplicon Sequence Variants (ASVs) this potential loss can be avoided due to all their properties such as ASVs inferred independently from different studies or different samples can be comparable across studies, reduced need for computation power, and are not limited by incomplete reference databases to mention some of them [43, 44]. ASVs allow individual experiment and the results could be tested and validated in separate datasets in contrast to merging datasets as in pooling analysis [29]. Using our methodology, we are able to achieve a signature of taxa across different datasets. In contrast with [51], where a signature of taxa between the microbiome and the diagnosis of ASD was not found through the analysis of various datasets. To the best of our knowledge, these type of experiments are not reported in the literature. The complete resulting taxa for each experiment is in Tables 1-3 of Additional file 1. Visualization of difference abundance of the results is in Supplementary figures 1-12 of Additional file 2. Finally, for individual AUC and MCC obtained in the Random Selection is in Additional file 3.

Despite the promising results and findings, more research and experimentation should be done with microbiome sequencing because counterexamples can be found that make this methodology ineffective. Such is the case with datasets related to asthma: PRJEB44044 [52], PRJNA601757 [53], and PRJNA913468 [54], where the feature selection and testing phase were inefficient. This was due to the lack of datasets with samples from the same source, the quality of the sequences, the lack of documentation, variations in the technical sequencing equipment used, also known as the *batch effect* [55, 56]. Thus, this methodology is dependent of the batch effect. Additionally, the experiments must be extended to study the relationship taxa-disease or taxa-disorder for possible medical applications.

Furthermore, from all experiments, it is easy to notice that the classification performance on the discovery dataset is considerably higher than those on the validation datasets. There are two possible explanations for this result. First of all, not all ASV features selected by the proposed methodology on the discovery dataset are found in the validation datasets: thus, the classifiers do not have access to all the information that led to the better performance on the discovery data, resulting in an decreased AUC and MCC. Secondly, the datasets could present differences due to the *batch effect*. We intentionally did not apply any batch correction methodology in this work, to better isolate and study the results of the proposed methodology.

## Conclusion

We developed a methodology for reproducible biomarker discovery for 16s rRNA microbiome sequence analysis, addressing the issues related with high dimensional data with a small number of samples, inconsistent results, the lack of uniform processing and analysis methodologies, and to achieve validations in separate databases. The results from the three experiments show that the proposed methodology achieved better performance (AUC and MCC) compared to K-Best and 10-time random selection methods. This methodology is a first approach to increase reproducibility, to provide robust and reliable results, and further testing needs to be done, as shown by the experiment in Asthma (PRJEB44044, PRJNA601757 and PRJNA913468) described in the discussion section. Nevertheless, the approach to the individual study of ASVs makes possible to identify small variations that can have a positive impact on medical applications. This methodology provides results that hopefully will allow pharmacologists, biologists, and health researchers to direct their efforts to the analysis of a list with a smaller number of individual taxa, instead of thousands of taxa grouped in clusters.

## Methods

### Methodology

**Fig. 4:**
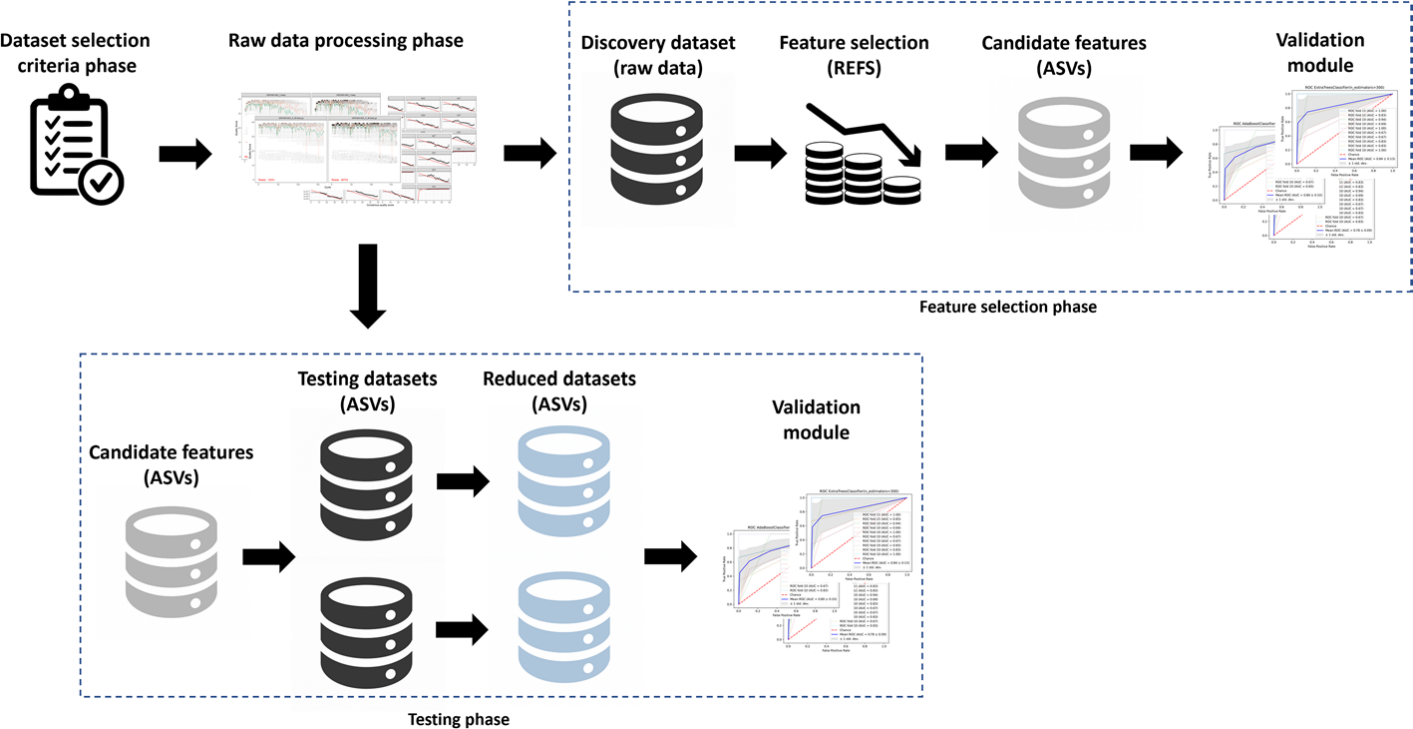
Overview of the proposed methodology. The upper shows the workflow for the dataset selection criteria, raw data processing and feature selection phases. The lower part shows the testing phase workflow

The proposed methodology consists of four phases: (1) dataset selection criteria, (2) raw data processing, (3) feature selection, and (4) testing. In contrast to other methodologies, such as pooling analysis [29, 51], we do not combine more than two datasets to produce a single one to be analyzed. The proposed methodology is oriented to work with Amplicon Sequence Variants (ASVs) because they can be used in independent studies [43, 44]. Using ASVs provide a possible solution to avoid inconsistent results, at the same time, they can help achieve external validation in separate datasets which [29]. For external validations, we recommend working with at least three datasets: one for discovery and the rest for testing. We address the issues of overfitting and biased performance associated with the datasets implementing a nested cross-validation scheme [42]. To provide a reproducible approach in the microbiome research, we document software versions, description about each phase and the necessary code/scripts to perform experiments are available on Github (https://github.com/steppenwolf0/MicrobiomeREFS). An overview of the proposed methodology is illustrated in Fig. 4.

The *dataset selection criteria* phase involves the selection, download, and extraction of relevant information from metadata (e.g., samples labels). The datasets must meet the following conditions:

- The databases should be 16s ribosomal RNA (rRNA) amplicon sequencing and belong to the same domain such as disease, disorder, or medication.
- There should be a minimum of two groups such as a control group and a case group.
- Each group should have a minimum of 10 samples.
- The documentation, whether it be metadata or a scientific paper, should clearly specify which group each sample belongs to.
- Datasets should have the same source of samples such as tissue, feces, or mucosa.

The *raw data processing phase* involves performing amplicon workflow on the raw data in the selected datasets and generate ASVs (features). We selected the DADA2 pipeline^1^ [25] due to its clear documentation. The DADA2 open-source R package allows to implement the full amplicon workflow on 16s rRNA sequences: filtering, dereplication, sample inference, chimera identification, and merging of paired-end reads [25]. We developed a DADA2-based script in R version 4.1.2, the code editor was RStudio version 2022.07.2 build 576, the DADA2 library version was 1.22.0, the DECIPHER library version was 2.22.0, the BiocManager library version was 1.30.19, and the taxonomy assignment was performed based on the SILVA_SSU_r138_2019^2^ reference database.

The *feature selection phase* aims to identify features, since we are working with the sequence as feature instead of the taxa because sequence is unique on the dataset, the feature selected should be contained in testing, so one dataset must be selected for discovery. The eligibility criteria for the discovery dataset is the one that contains the shortest sequence length after the raw data processing phase. Once the discovery dataset is selected, we have to perform two processes:

1. The Recursive Ensemble Feature Selection (REFS),which is an algorithm for identifying biomarkers by determining the features that are most effective in differentiating between groups in datasets achieving the highest accuracy with the fewest number of features [48, 57–62]. The ensemble is composed by 8 classifiers from the scikit-learn toolbox [63]: Stochastic Gradient Descent (SGD) on linear models, Support Vector Machine classifier (SVC), Gradient Boosting, Random Forest, Logistic Regression, Passive Aggressive classifier, Ridge Classifier and Bagging. To minimize overfitting and biased performance, REFS employs a nested approach within a 10-fold cross-validation scheme, which is a proven solution to yield more accurate and unbiased results, even with a small sample size [42]. REFS was built on python version 3.10.8 using the scikit-learn toolkit version 1.1.3.
2. Validation, to minimize bias selection, we developed a validation module with 5 different classifiers from the scikit-learn toolkit [63]: AdaBoost, Extra Trees, KNeighbors, Multilayer Perceptron (MLP), and LassoCV. This validation module also employs a nested approach within a 10-fold cross-validation scheme. This module must be executed two times: (1) using samples labels, the selected features, and the corresponding abundance, and (2) using samples labels, all features, and the corresponding abundance. The 5 classifiers provides an average value for the area under curve (AUC), that evaluates the effectiveness of a discriminant test. Values approaching to 1.0 indicate excellent performance [50].

These processes should be executed at least 10 times concurrently, to compensate for the stochasticity of some of the classifiers used in the study (e.g. Random Forest) and the internal cross-validation process.

The *testing phase* involves testing the features selected by using REFS in a minimum of two separate datasets. The selected features must be searched on each testing dataset. Features can be repeated in the testing datasets, so we must follow the next process: if *Feature x* is present n-times in the testing dataset, the final abundance of *Feature x* will be the sum of the abundance of those n-occurrences. To validate the features found in each testing dataset, the validation module must be executed one time on each testing dataset using as input the samples labels, the found features, and the corresponding abundance. The AUC is employed as a measure of diagnostic accuracy.

Additionally, we conducted a comparative analysis with two different feature selection methods:

- *K-Best with F-score*. This selection method will be applied to the discovery dataset instead of REFS. We used the SelectKbest algorithm from the scikit-learn toolbox which selects the K top-scoring features based on a user-defined metric, with the F-score [63]. The value assigned to K is determined by the number of features obtained using REFS. For instance, if REFS selected 10 features, the value of K would be set to 10.
- *10-time random selection*. This method consists in randomly select a given number of features from all features in each testing dataset. This given number is determined by the number of features found in each testing dataset. For instance, if 8 out of 10 features selected by using REFS were identified in the testing dataset, then 8 features will be randomly selected each time.

The AUC provided by the validation module is used as a metric for comparing the results of the proposed methodology with these two feature selection methods. Additionally, we use the Matthews Correlation Coefficient (MCC) [64] as a metric to evaluate the performance of the methodology as well as for comparison with other feature selection methods.

### Datasets

**Fig. 5:**
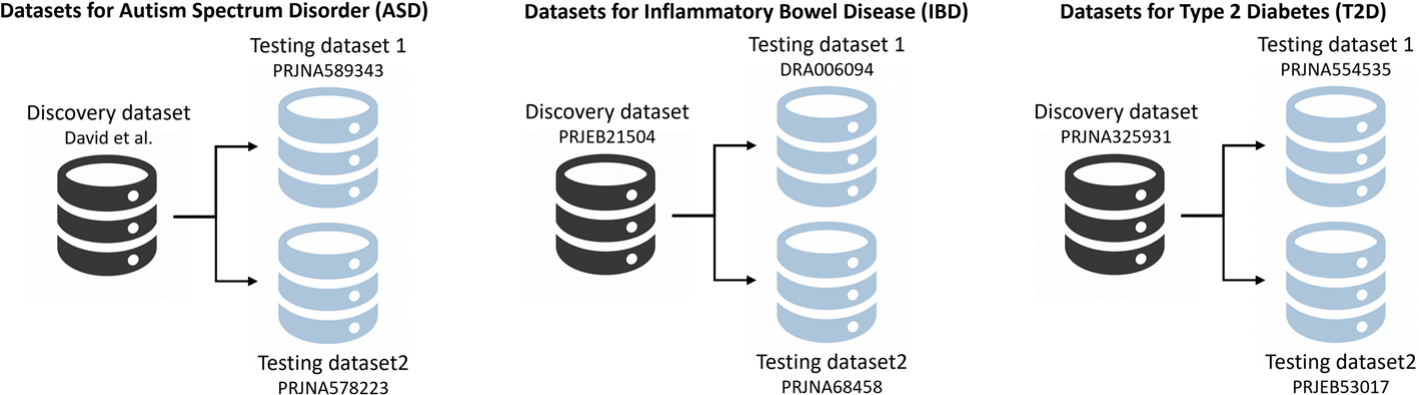
Overview of the datasets used for each experiment

We used a total of nine datasets, with three datasets for each experiment: Autism Spectrum Disorder (ASD), Inflammatory Bowel disease (IBD), and Type 2 Diabetes (T2D), see Fig. 5. Each dataset adhered to the data selection criteria phase. We considered only two groups within each dataset: control and cases. The control group is made up of healthy people or people in remission, the case group is made up of people diagnosed with the medical condition.

The datasets related with *ASD* are: (1) David et al [49]^3^ it has 117 samples of which 57 belong to the control group and 60 to the case group, (2) PRJNA589343 [65] downloaded from the NCBI public repository,^4^ it has 127 samples of which 50 belong to the control group and 77 to the case group, and (3) PRJNA578223 [66] downloaded from the NCBI public repository, it has 96 samples of which 48 belong to the control group and 48 to the case group.

The datasets related with *IBD* are: (1) PRJEB21504 [67] downloaded from the NCBI public repository, it has 95 samples of which 66 belong to the control group and 29 to the case group, (2) DRA006094 [68] downloaded from the NCBI public repository, it has 70 samples of which 15 belong to the control group and 55 to the case group, and (3) PRJNA68458  [69] downloaded from the NCBI public repository, it has 103 samples of which 45 belong to the control group and 58 to the case group.

The datasets related with *T2D* are: (1) PRJNA3259311 [70] downloaded from the NCBI public repository, it has 112 samples of which 84 belong to the control group and 28 to the case group, (2) PRJNA5545355 [71] downloaded from the NCBI public repository, it has 60 samples of which 20 belong to the control group and 40 to the case group, and (3) PRJEB53017 [72] downloaded from the NCBI public repository, it has 94 samples of which 46 belong to the control group and 48 to the case group.

### Supplementary information

**Additional file 1.** The complete resulting taxa for each experiment is in Supplementary Table 1–3.

**Additional file 2.** Visualization of difference abundance of the results is in Supplementary Figures 1–12.

**Additional file 3.** Individual AUCs and MCCs for each random validation in the 3 experiments.

## Data Availability

The code/scripts to perform experiments are available on Github (https://github.com/steppenwolf0/MicrobiomeREFS).

## Footnotes

1 DADA2 pipeline is available in https://benjjneb.github.io/dada2/tutorial_1_8.html.

2 Available in: http://www2.decipher.codes/Classification/TrainingSets/.

3 Dataset available in: http://files.cgrb.oregonstate.edu/David_Lab/ASD_study1/,

4 https://www.ncbi.nlm.nih.gov/.
