# Supplementary material for: Methodology for biomarker discovery with reproducibility in microbiome data using machine learning
Source: BMC Bioinformatics. 2024 Jan 15;25:26. doi: 10.1186/s12859-024-05639-3 (PMC10789030; doi:10.1186/s12859-024-05639-3)
Supplement: Supplementary file 2 — Additional file 2. Visualization of difference abundance of the results is in Supplementary Figures 1–12. [file 12859_2024_5639_MOESM2_ESM.docx]

**Supplementary figures for ASD**

**
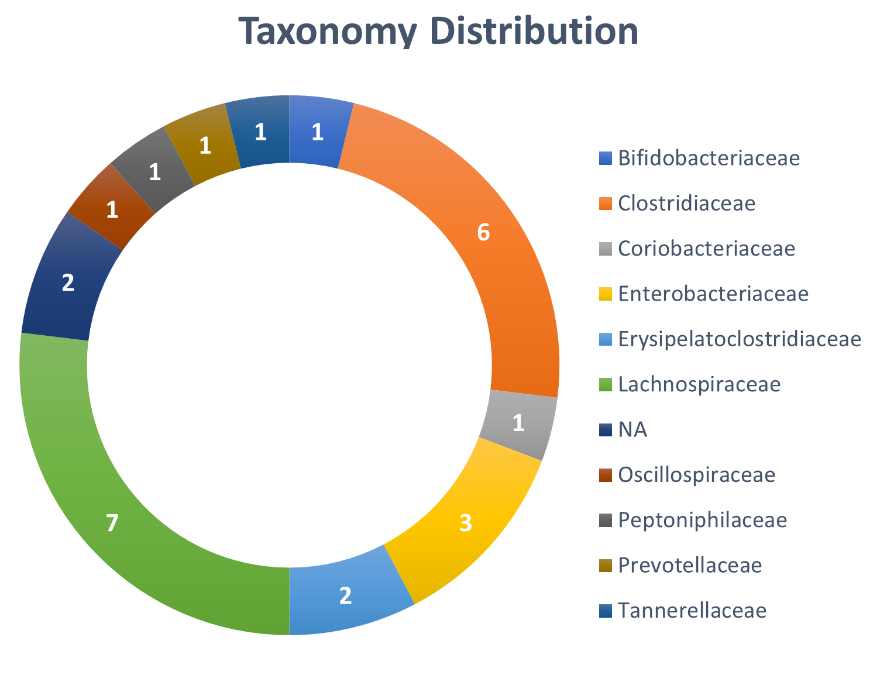
**

**Supplementary figure 1.** Family-level taxonomy distribution of the 26 features selected by using REFS.


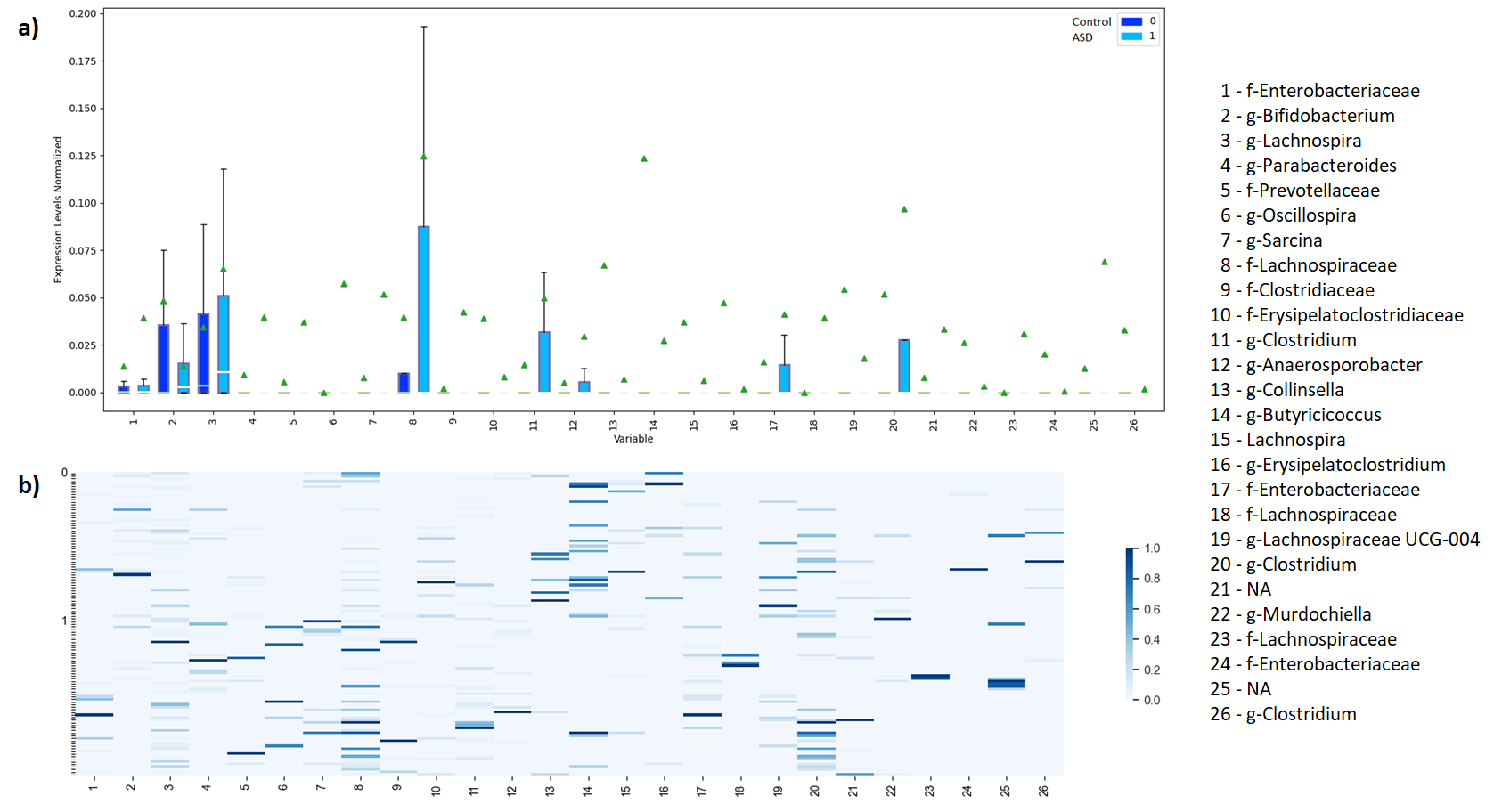


**Supplementary figure 2.** a) Boxplot representing differential abundance means (dark and light bars) and medians (green triangle) of the selected 26 features from the discovery dataset. b) Heatmap with the differential abundances for each selected feature from the discovery dataset. Each feature is named using the following format: Taxonomy Level - Name. The value ‘NA’ indicates that the feature was not identified.


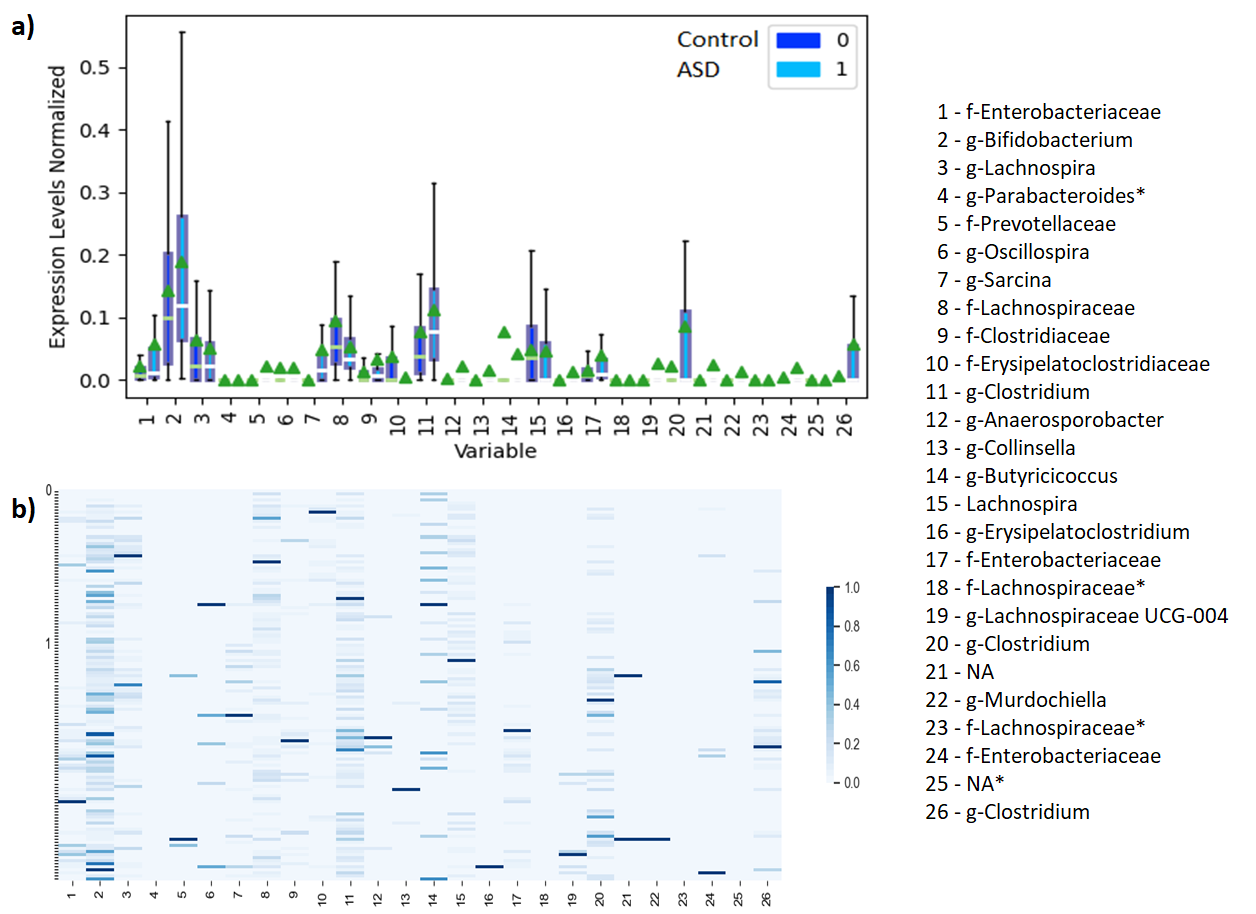


**Supplementary figure 3.** a) Boxplot representing differential abundance means (dark and light bars) and medians (green triangle) of the 22 of 26 features found in PRJNA589343. b) Heatmap with the differential abundances for each feature found in PRJNA589343. Each feature is named using the following format: Taxonomy Level - Name. The value ‘NA’ indicates that the feature was not identified. ‘*’ indicates that the feature was not found in this dataset.

**
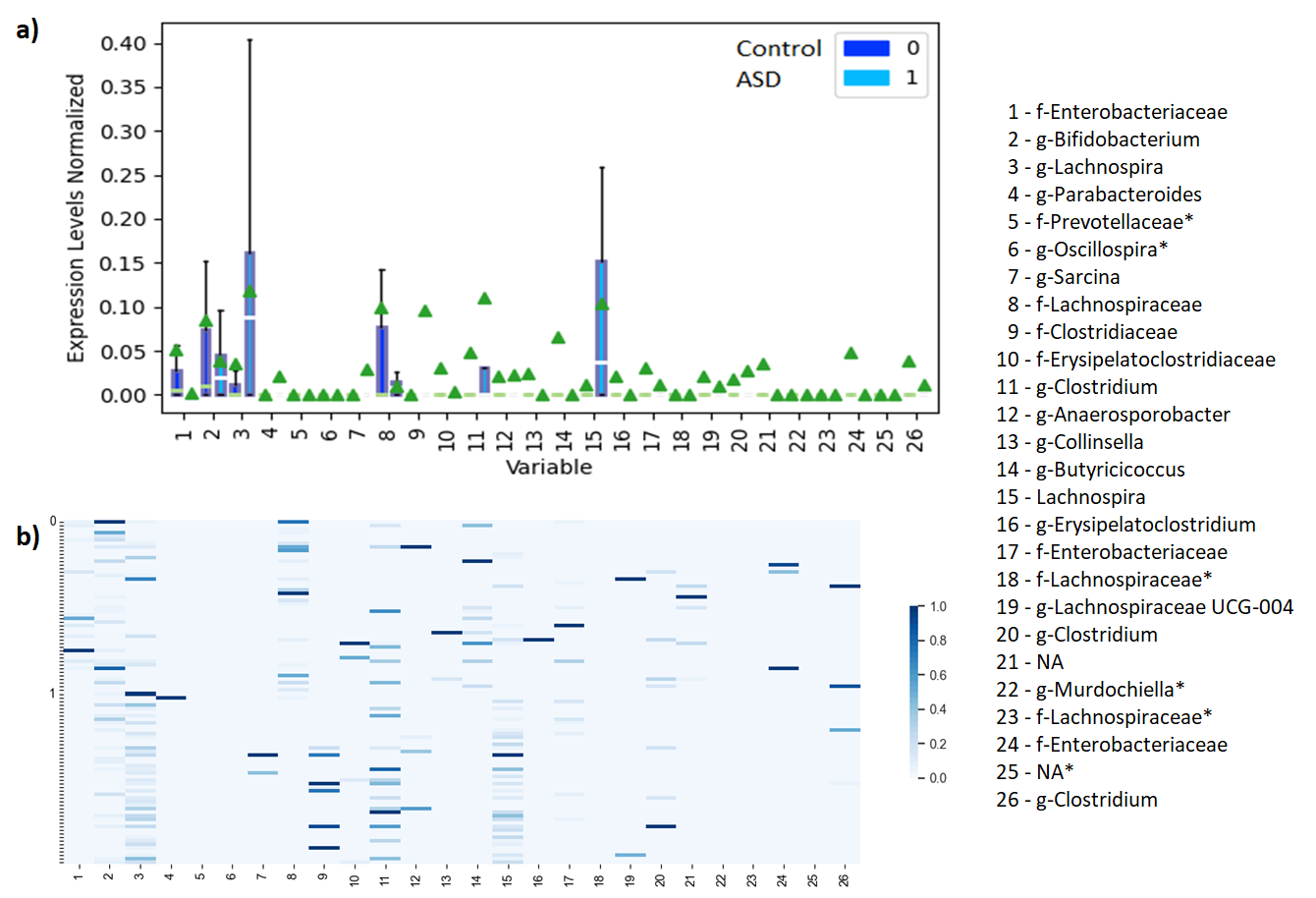
**

**Supplementary figure 4.** a) Boxplot representing differential abundance means (dark and light bars) and medians (green triangle) of the 20 of 26 features found in PRJNA578223. b) Heatmap with the differential abundances for each feature found in PRJNA578223. Each feature is named using the following format: Taxonomy Level - Name. The value ‘NA’ indicates that the feature was not identified. ‘*’ indicates that the feature was not found in this dataset.

**Supplementary figures for IBD**

**
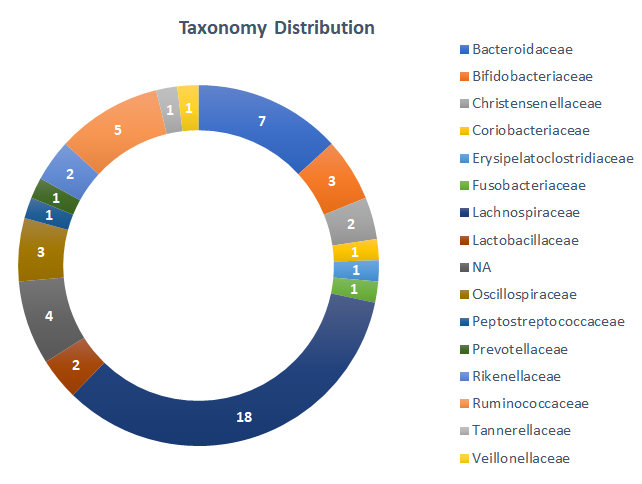
**

**Supplementary figure 5.** Family-level taxonomy distribution of the 53 features selected by using REFS.


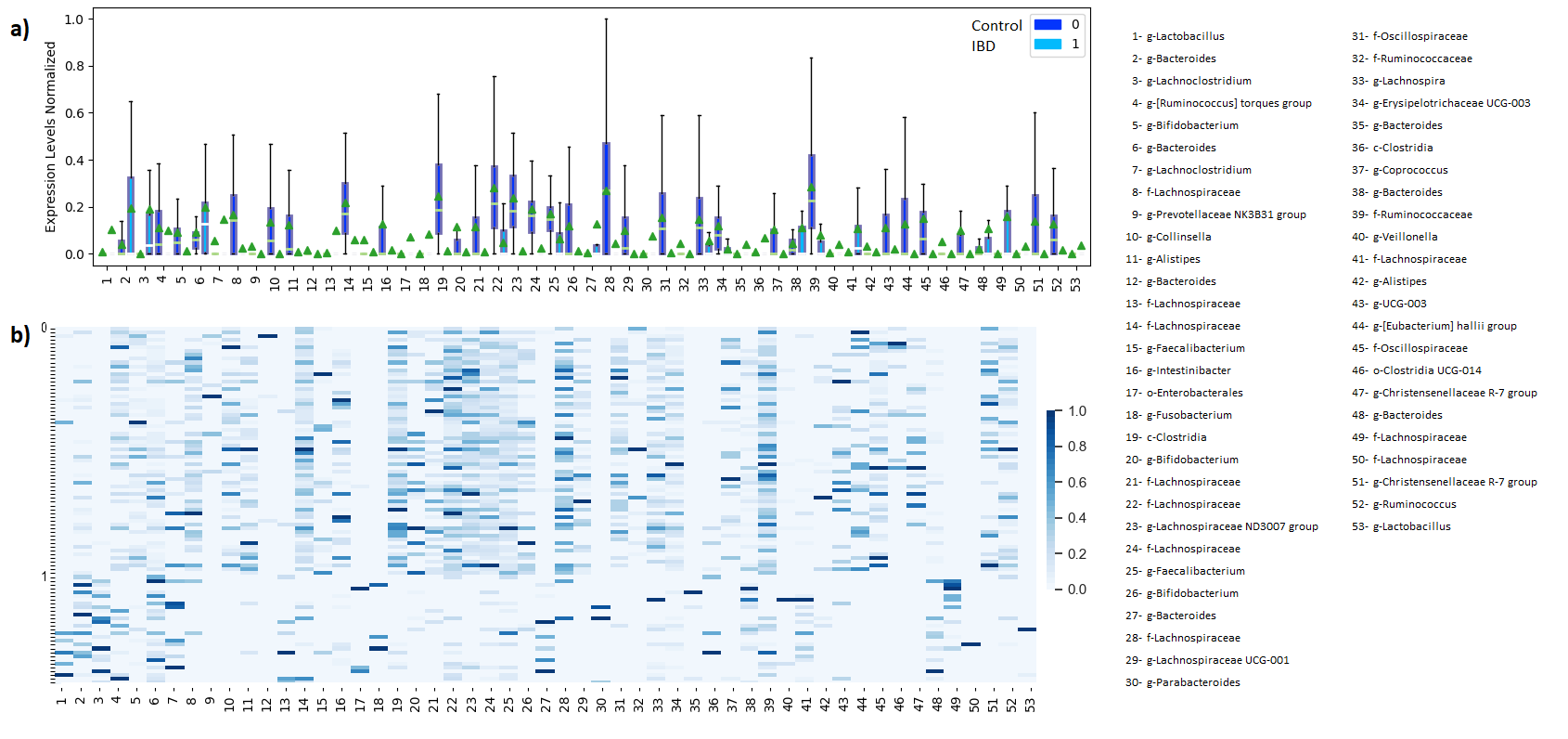


**Supplementary figure 6.** a) Boxplot representing differential abundance means (dark and light bars) and medians (green triangle) of the selected 53 features from the discovery dataset. b) Heatmap with the differential abundances for each selected feature from the discovery dataset. Each feature is named using the following format: Taxonomy Level – Name.


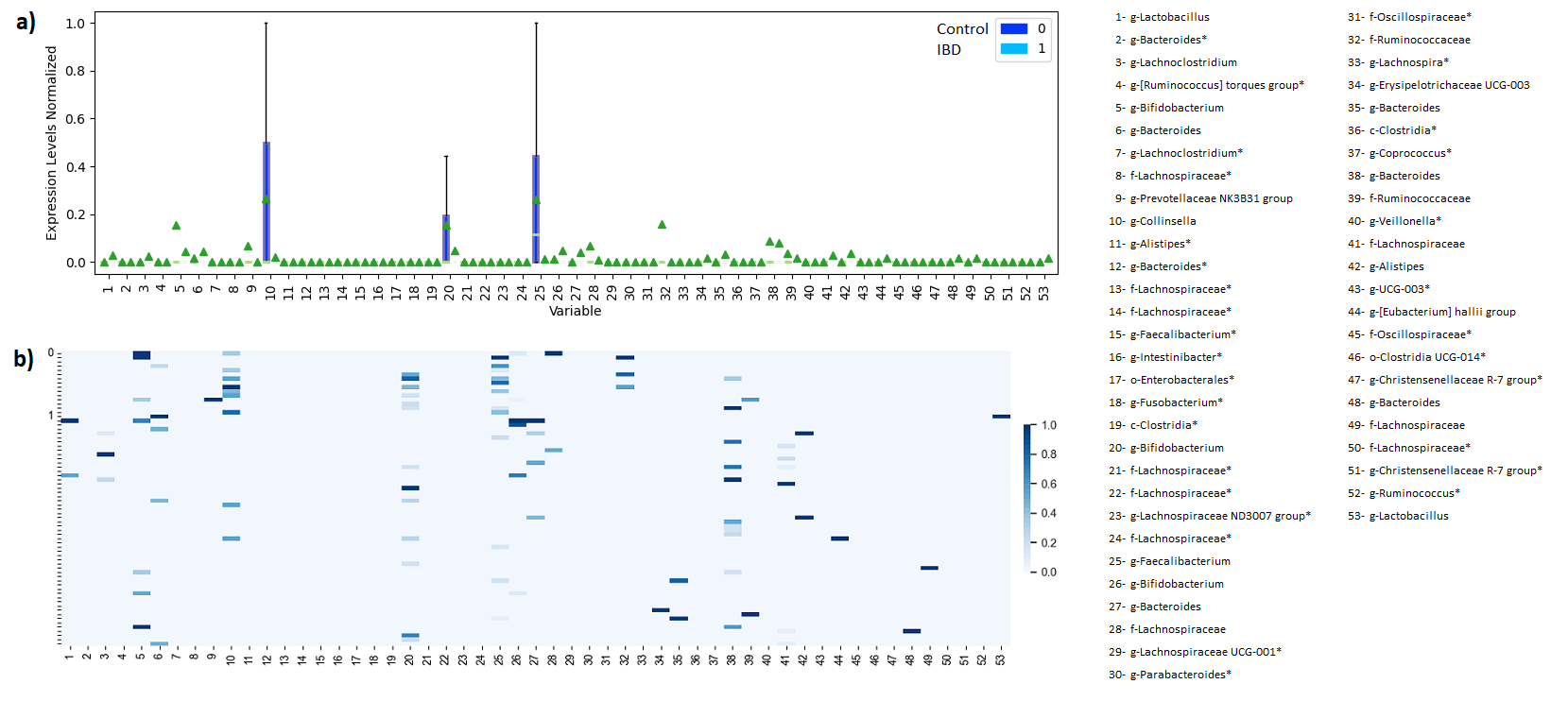


**Supplementary figure 7.** a) Boxplot representing differential abundance means (dark and light bars) and medians (green triangle) of the 22 of 26 features found in DRA006094. b) Heatmap with the differential abundances for each feature found in DRA006094. Each feature is named using the following format: Taxonomy Level - Name. The value ‘NA’ indicates that the feature was not identified. ‘*’ indicates that the feature was not found in this dataset.


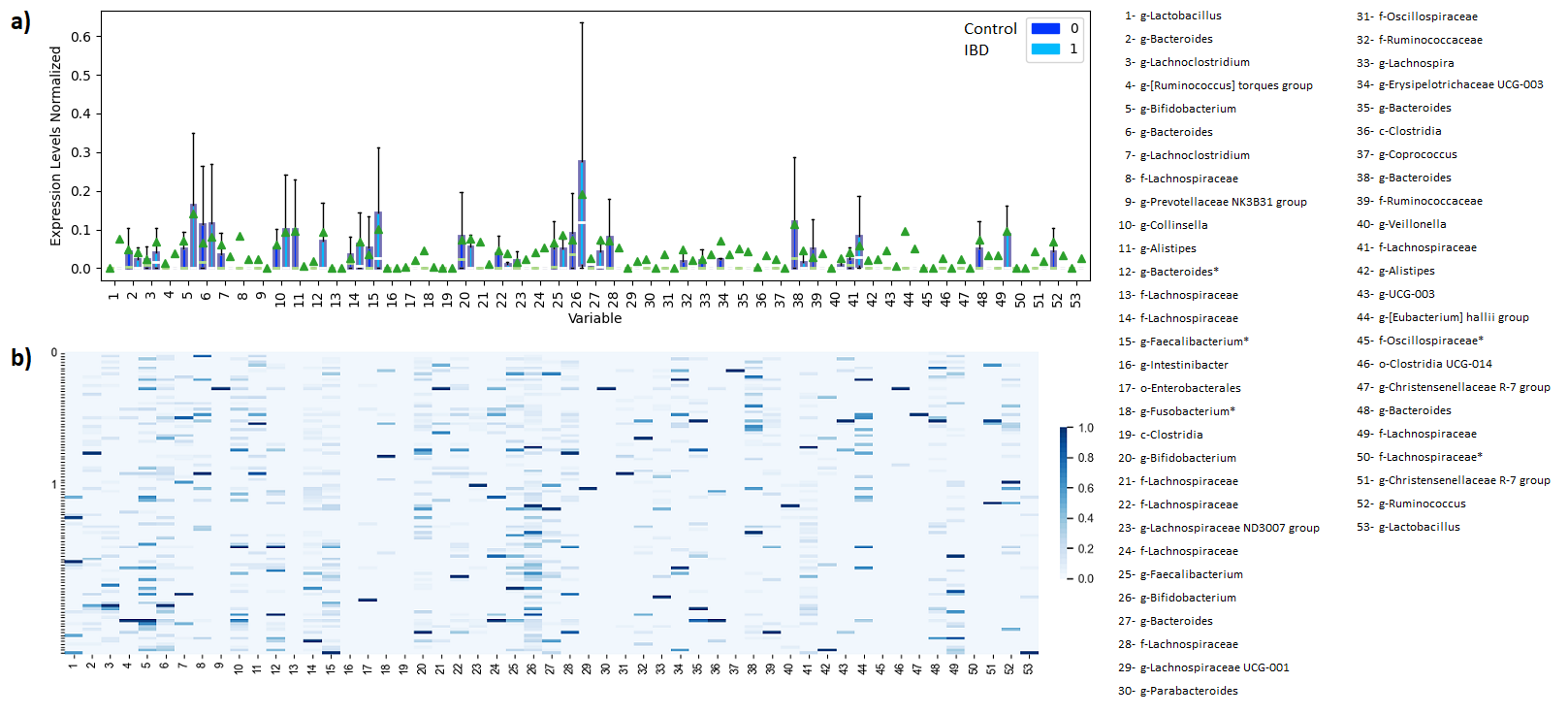


**Supplementary figure 8.** a) Boxplot representing differential abundance means (dark and light bars) and medians (green triangle) of the 20 of 26 features found in PRJNA684584. b) Heatmap with the differential abundances for each feature found in PRJNA684584. Each feature is named using the following format: Taxonomy Level - Name. The value ‘NA’ indicates that the feature was not identified. ‘*’ indicates that the feature was not found in this dataset.

**Supplementary figures for T2D**

**
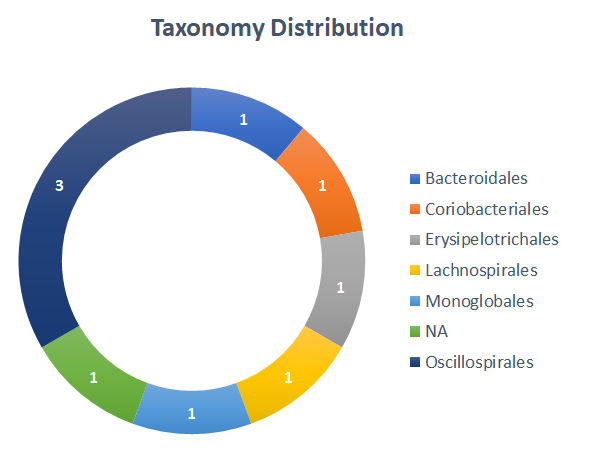
**

**Supplementary figure 9.** Order-level taxonomy distribution of the 9 features selected by using REFS.


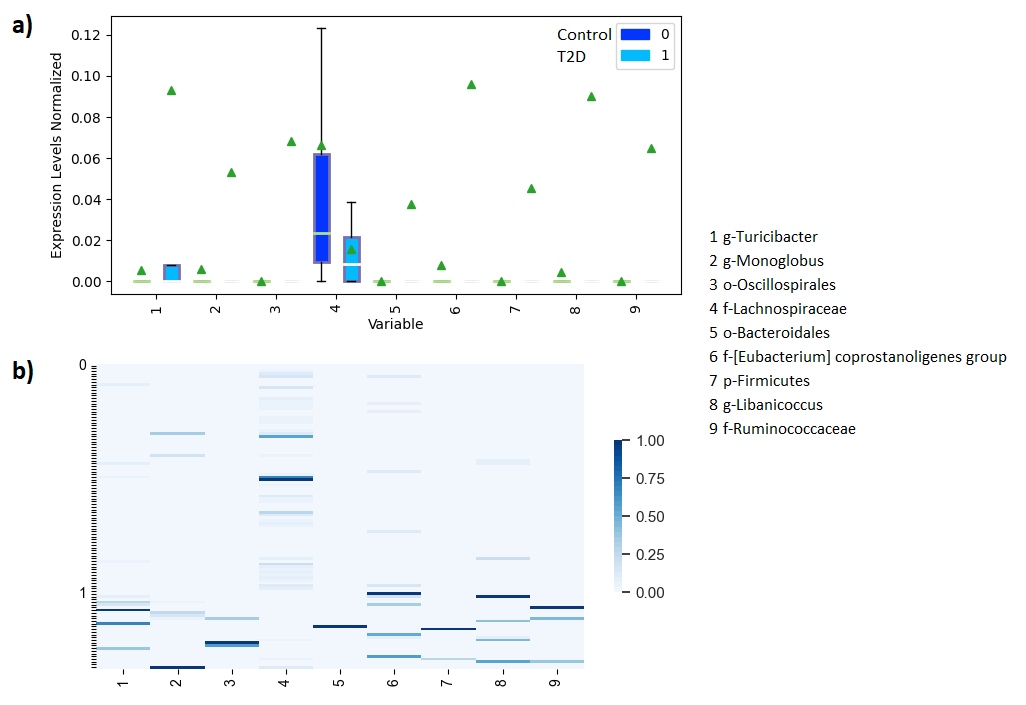


**Supplementary figure 10.** a) Boxplot representing differential abundance means (dark and light bars) and medians (green triangle) of the selected 9 features from the discovery dataset. b) Heatmap with the differential abundances for each selected feature from the discovery dataset. Each feature is named using the following format: Taxonomy Level - Name.

**
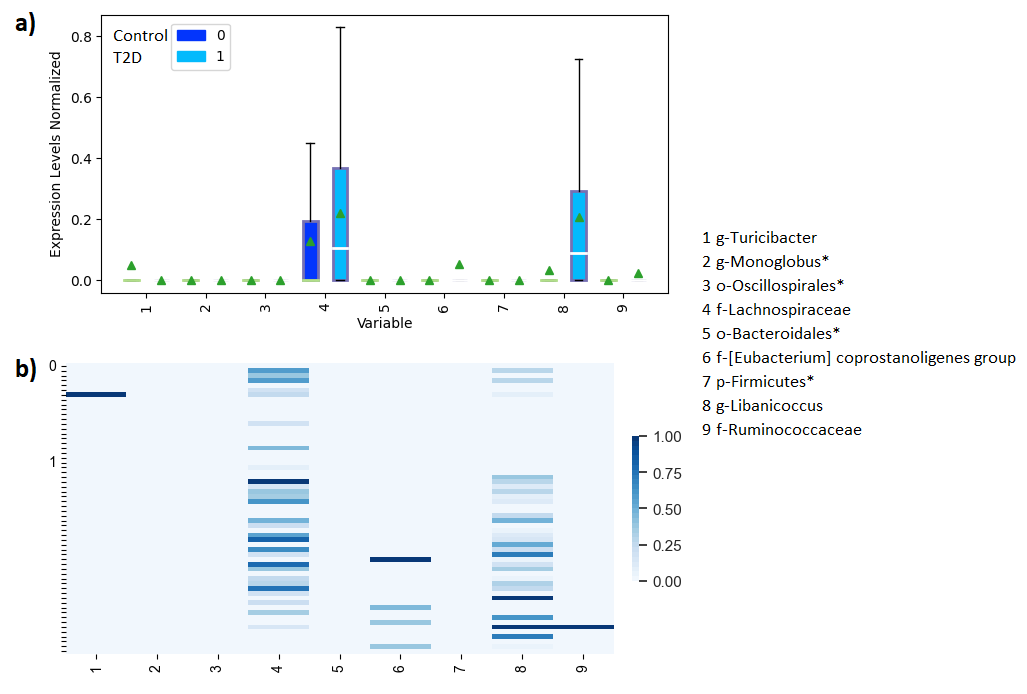
**

**Supplementary figure 11.** a) Boxplot representing differential abundance means (dark and light bars) and medians (green triangle) of the 5 of 9 features found in PRJNA554535. b) Heatmap with the differential abundances for each feature found in PRJNA554535. Each feature is named using the following format: Taxonomy Level - Name. The value ‘NA’ indicates that the feature was not identified. ‘*’ indicates that the feature was not found in this dataset.


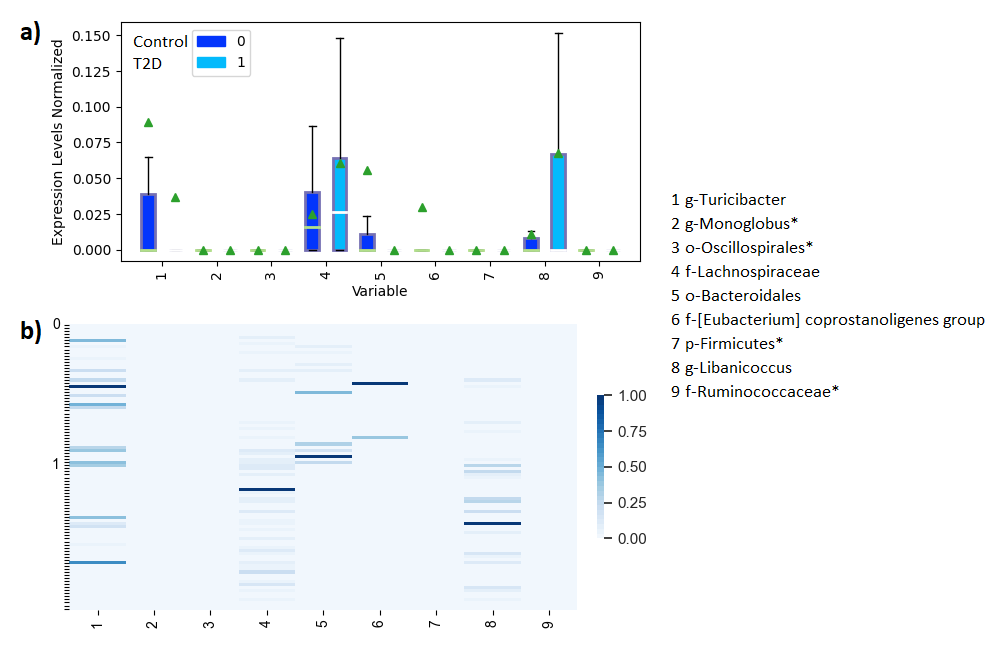


**Supplementary figure 12**. a) Boxplot representing differential abundance means (dark and light bars) and medians (green triangle) of the 5 of 9 features found in PRJEB53017. b) Heatmap with the differential abundances for each feature found in PRJEB53017. Each feature is named using the following format: Taxonomy Level - Name. The value ‘NA’ indicates that the feature was not identified. ‘*’ indicates that the feature was not found in this dataset.
